# Asthma risk in young people who attended chlorinated swimming pools in early childhood

**DOI:** 10.1590/1984-0462/2025/43/2024251

**Published:** 2025-08-18

**Authors:** Cristiano de Lima Silvestre, Ricardo Freitas-Dias, Emília Chagas Costa, Marcos André Moura dos Santos, Jânio Luiz Correia, Décio Medeiros Peixoto, Edil de Albuquerque Rodrigues, Marco Aurélio de Valois Correia

**Affiliations:** a Universidade de Pernambuco, Programa de Pós-Graduação em Hebiatria – Recife (PE), Brazil.; b Universidade Federal de Pernambuco, Programa de Pós-Graduação em Nutrição – Recife (PE), Brazil.; c Universidade Federal de Pernambuco, Centro de Pesquisas em Alergia e Imunologia, Hospital das Clínicas – Recife (PE), Brazil.; d Universidade Federal de Pernambuco, Núcleo de Educação Física e Ciências do Esporte – Vitória de Santo Antão (PE), Brazil.

**Keywords:** Swimming, Child, Asthma, Chlorine, Swimming pool, Natação, Criança, Asma, Cloro, Piscinas

## Abstract

**Objective:**

The aim of this study was to evaluate the association between exposure to volatile chlorination by-products from swimming during early childhood and the subsequent development of asthma

**Data source:**

This systematic review was conducted in accordance with the Preferred Reporting Items for Systematic Reviews and Meta-Analyses (PRISMA) guidelines. The review protocol was registered in the International Prospective Register of Systematic Reviews (PROSPERO) under CRD42021291850. Searches were carried out in the following electronic databases: PubMed, Cochrane Library, Google Scholar, and ScienceDirect, using the terms "children in early childhood," "swimming in a chlorinated pool," "exposed and unexposed," and "asthma risk," covering studies published between 2003 and 2020. The review included cohort and cross-sectional studies of individuals who swam in chlorinated pools during early childhood. The methodological quality of the studies was evaluated for risk of bias.

**Data synthesis:**

Out of 6865 studies screened, six met the inclusion criteria, involving 4058 subjects and 310 asthma events (2365 in the exposure group and 1693 in the control group). The combined effect, expressed as an odds ratio for asthma risk in all included studies, was 1.09 (95%CI 0.67–1.77; p=0.740).

**Conclusions:**

This is the first systematic review to comprehensively evaluate the association between swimming in chlorinated pools and the risk of developing asthma. No association was found between childhood swimming in chlorinated pools and the risk of asthma development.

## RESUMO

**Objetivo:**

Avaliar a associação entre a exposição a produtos voláteis da cloração de piscinas durante a natação na primeira infância e o desenvolvimento subsequente de asma.

**Fonte de dados:**

Esta revisão sistemática foi conduzida de acordo com as diretrizes do *Preferred Reporting Items for Systematic Reviews and Meta-Analyses (PRISMA)*. O protocolo da revisão foi registrado no *International Prospective Register of Systematic Reviews (PROSPERO)* sob o número CRD42021291850. As buscas foram realizadas nas seguintes bases de dados eletrônicas: *PubMed, Cochrane Library, Google Scholar e ScienceDirect*, utilizando os termos "crianças na primeira infância", "natação em piscina clorada", ";expostos e não expostos" e ";risco de asma", abrangendo estudos publicados entre 2003 e 2020. Foram incluídos estudos de coorte e transversais envolvendo indivíduos que nadaram em piscinas cloradas durante a primeira infância. A qualidade metodológica dos estudos foi avaliada quanto ao risco de viés.

**Síntese dos dados:**

Dos 6.865 artigos avaliados, seis atenderam aos critérios de inclusão, envolvendo 4.058 indivíduos e 310 casos de asma (2.365 no grupo de exposição e 1.693 no grupo controle). O efeito combinado, expresso como *odds ratio* para o risco de asma em todos os estudos incluídos, foi de 1,09 (IC95% 0,67–1,77; p=0,740).

**Conclusões:**

Esta é a primeira revisão sistemática a avaliar de forma abrangente a associação entre a natação em piscinas cloradas e o risco de desenvolvimento de asma. Não foi encontrada associação entre a natação em piscinas cloradas na infância e o risco de desenvolvimento de asma.

## INTRODUCTION

 Swimming has been one of the most practiced sports worldwide since ancient times, recognized for its ability to develop physical skills and promote harmonious body movement, while offering benefits such as muscle strengthening and optimization of cardiovascular and respiratory systems.^1-4^ People engage in swimming not only to acquire the skill but also to enhance their overall quality of life.^3,5,6^

 Chlorine compounds used to disinfect swimming pools react with organic matter, such as urine and sweat, producing by-products like trihalomethanes that can be inhaled and potentially harm human health.^7,8^ Despite this, chlorine and its derivatives have been deemed safe for pool disinfection.^2,4^ Researchers increasingly associate swimmers’ respiratory health deterioration with the irritating effects of chlorine or its by-products. These compounds can be inhaled as gases, microaerosols, or small volumes of water, particularly by infants during swimming.^9-12^ It has been hypothesized that these chemicals irritate swimmers’ airways, increasing sensitivity to environmental stressors such as allergens or infectious agents.^7,13-16^

 Thus, regular exposure to chlorinated pools in infancy may be causally linked to potential respiratory health repercussions in children.^1,3,8,12-18^ Environmental exposures, including volatile chlorination products, may affect the lung epithelium and increase asthma incidence; however, data remain sparse and inconclusive.^1,3,8,12-17^ Therefore, this study aimed to evaluate the association between exposure to volatile chlorination products from swimming in early childhood and the subsequent development of asthma, thereby advancing knowledge in this important area. 

## METHOD

### Protocol and registration

 This systematic review was conducted in accordance with the recommendations of the Preferred Reporting Items for Systematic Reviews and Meta-Analyses (PRISMA).^19^ The review protocol was registered in the International Prospective Registry of Systematic Reviews (PROSPERO) [Centre for Reviews and Dissemination, York, UK. Available at: www.crd.york.ac.uk/PROSPERO], under registration CRD42021291850. 

### Eligibility criteria

 Studies had to meet the following criteria to ensure inclusion:

1. Cross-sectional or cohort studies with full text available in English,
2. Complete analytical study design with an appropriate control group,
3. Inclusion of individuals of both genders,
4. Exposure criteria — swimming in a chlorinated pool in early childhood, and
5. Outcome criteria — asthma classification.

The following were excluded:

1. Review studies,
2. Editorials,
3. Conference publications,
4. Theses/dissertations,
5. Randomized controlled trials,
6. Children with asthma at the time of exposure,
7. Lack of information regarding the swimming period or the swimming-asthma relationship,
8. *In vitro* studies and accidental exposure,
9. Case reports,
10. Studies without a control group,
11. Studies with incomplete design (*e.g*., ecological studies), and
12. Assessment of workers and professional swimmers.

### Search strategy

 Comprehensive searches were conducted in English across the following electronic databases:

1. PubMed;
2. Cochrane Library;
3. Google Scholar; and
4. ScienceDirect, covering studies published between 2003 and 2020.

Searches were performed using descriptors from Medical Subject Headings (MeSH). The Population, Exposure, Comparison and Outcomes (PECO) strategy was employed, as recommended by The Cochrane Handbook for Systematic Reviews of Interventions, using the terms related to children in early childhood, swimming in a chlorinated pool; exposed and unexposed individuals, and asthma risk. These terms were combined using the Boolean operator "AND," resulting in four searches performed in the databases (Table 1). The search strategies were developed by an expert researcher (C.D.L.S.), with a second expert researcher (E.A.R.F.) conducting the review, following the Peer Review of Electronic Search Strategies (PRESS) guideline. 

**Table 1:** Database search strategy.

Mean (SD)	Minimum–Maximum
1) PubMed 2) Cochrane Library 3) Google Scholar 4) ScienceDirect	#1 (infant) AND (swimming) #2 (infant) AND (swimming) AND (asthma) #3 (infant) AND (swimming AND wheezing #4 baby AND swimming #5 baby AND swimming AND asthma #6 baby AND swimming AND wheezing #7 #1 AND #2 AND #3 AND #4 AND #5 AND #6

### Study selection

 Duplicate studies were identified and retrieved by a trained researcher (C.D.L.S.) and exported to Microsoft Excel 2010 (Microsoft Excel software; Microsoft Corporation, WA, USA). The studies identified in the search were selected by two independent reviewers (C.D.L.S. and E.A.R.F.) in two steps: 1) title and abstract screening; and 2) full-text reading. Studies were coded as "yes", "no" or "maybe" regarding eligibility.^20^

 In the first step, titles and abstracts were examined to identify potentially eligible studies. If at least one reviewer considered a study eligible, the full text was obtained for evaluation. Full-text articles were assessed for eligibility using the criteria outlined above, with reasons for exclusions documented. Disagreements were discussed until a consensus was reached, and a third researcher (M.A.V.C.J) was consulted if necessary. 

 Additionally, a cross-reference search was performed by two independent researchers using the reference lists of the included studies to identify relevant studies not captured in the electronic search. The authors of eligible studies were contacted via email when confirmation of data or additional information was required. 

### Data extraction

 Two evaluators (C.D.L.S. and E.A.R.F.) independently extracted data from published articles using a standard form that included the following information: authors, year of publication, place of publication, study type, sample size, cohort period, outcome evaluation method, and outcome prevalence. Data from studies meeting the eligibility criteria were entered into to Microsoft Excel 2010®. 

### Risk of bias

 The methodological quality of the studies was evaluated using the Cochrane’s Bias Methods Group guidelines, as per The Cochrane Handbook for Systematic Reviews of Interventions.^20^

 The assessment includes eight items:

1. Selection of exposed and non-exposed cohorts;
2. Confidence in exposure assessment;
3. Confident in outcome assessment;
4. Matching of exposed and unexposed groups for all variables;
5. Confidence in assessing prognostic factors;
6. Confidence in outcome assessment;
7. Follow-up of cohorts; and
8. Similarity of interventions between groups. Each criterion is classified as "definitely yes," "probably yes," "probably not," or "definitely no."

Disagreements were discussed until a consensus was reached, and a third or fourth researcher (R.F.D. and M.A.V.C.J.) was consulted if necessary. 

### Statistical analysis

 Statistical analyses and meta-analysis were conducted using the RStudio program (RStudio software; RStudio, PBC, Boston, MA, USA) with the Mantel-Haenszel method.^21^ The analyzed database includes results from studies where children aged 0 to 3 years were exposed to swimming in chlorinated pools (regular practice of swimming in open and closed chlorinated pools, regardless of frequency and duration) *versus* non-swimmers. A random-effects model, Peto analysis method, and odds ratio (OR) with 95% confidence intervals (CI) for dichotomous outcomes were utilized. Forest plots were visually inspected for effect direction and magnitude, with OR>1 and a 95%CI not overlapping the null value indicating a higher likelihood of asthma with pool attendance. Statistical heterogeneity was assessed using the Cochrane’s Q test (significance level, p<0.10) and the I2 test: <25% (low), <50% (moderate), >75%, (high).^22^

 Outcome variables selected for meta-analysis included "physician-diagnosed asthma," "asthma identified by questionnaires," or "asthma diagnosed by pulmonary function tests." 

## RESULTS

**Figure 1:**
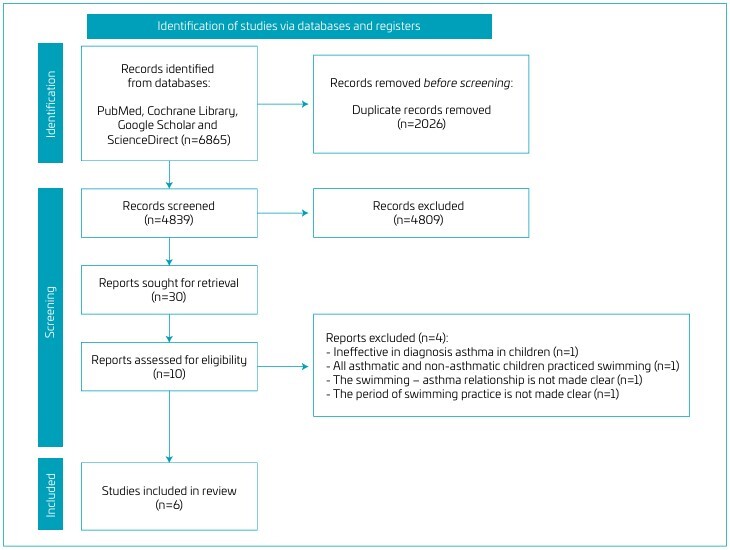
Study selection flowchart.

Figure 1 presents the flowchart of the research screening process, which yielded 6865 titles from the initial search. After removing duplicates and screening titles and abstracts, 30 articles were selected for full review and analysis, of which 10 were considered potentially relevant for this review. An in-depth analysis of these 10 articles led to the exclusion of four publications for the following reasons: lack of information on asthma diagnosis in children (n=1);^17^ both asthmatic and non-asthmatic children practiced swimming (n=1);^8^ the relationship between swimming and asthma was not clearly defined (n=1);^7^ and the period of swimming practice was not specified (n= 1).^23^

 Additionally, two studies were excluded from the meta-analysis because they did not separately present the number of participants in the swimming and control groups,^1^ and another due to missing data on the control group.^15^

Table 2 reports the characteristics of the remaining studies. Two prospective cohort studies and four cross-sectional studies were included. Three studies reported wheezing or coughing at the beginning of the investigation.^2,15,16^ The exposure period (swimming) for all studies occurred during early childhood (between 0 and 3 years), and the outcome analysis (asthma) ranged from 6 months to 13 years of age. The instruments used to assess respiratory and swimming symptoms, as well as the controlled variables, are also shown in Table 2. 

**Table 2:** Sample characteristics.

Author (Country)	Wheezing baseline	Exposure period	Swimmers/control (sample)	End period	Respiratory symptom assessment	Controlled variables
Nystad et al.^1^ (Norway)	No	0–6 month	2106/5813	6–18 month	Has your child had an episode of congestion or wheezing in the last 12 months?	Common variables, parity, season of childbirth, and daycare attendance.
Nystad et al.^1^ (Norway)	No	0–6 month	5783/17.168	6–18 month		
Schoefer et al.^2^ (Germany)	Yes	0–1 year	1279/913	6 years	NE	Common variables, study center, and day care.
Irahara et al.^16^ (Japan)	Yes	3 years	126/971	3–5 years	ISAAC	Common variables, household income, maternal age at birth, pet ownership at age 3, weekend television viewing at age 3, BMI at age 3.
Voisin et al.^14^ (Belgium)	No	0–2 years	195/235	5–6 years	ISAAC	Common variables, season of birth, cleaning the house with bleach, day care, childhood in urban or rural areas, residing in the vicinity of a polluting industry or within 100 m of a busy road, and the cumulative time spent in indoor or outdoor chlorinated pools before 2 years.
Bernard et al.^18^ (Belgium)	No	0–1 year	43/298	10–13 years	NE	Common variables, total and specific IgE for aeroallergens, housing density, pets, accumulated frequency in a chlorinated pool and access to a chlorinated home pool.
Font-Ribera et al.^14^ (Spain)	Yes	0–2 years	607/2758	6–12 years	ISAAC	Common variables and BMI.

Common variables refer to parental asthma/allergy, parental smoking, maternal age, gender, maternal education, birth weight, breastfeeding, number of siblings; exposure: childhood swimming; outcome: asthma.

NE: not specified; ISAAC: International Study of Asthma and Allergies in Childhood; BMI: body mass index.

Table 3 summarizes the authors’ results and conclusions on the association between early swimming and the development of asthma-related respiratory symptoms, along with the instruments used to classify outcomes. None of the articles explicitly concluded that swimming increases the risk of asthma. 

**Table 3:** Results and conclusions on the association between early swimming and the development of asthma-related respiratory symptoms.

Author	Swimmers n (%)	Control n (%)	Gross OR/RR (95%CI)	Adjusted OR/RR (95%CI)	Diagnosis	Conclusion
Nystad et al.^1^	NE (47.3)	NE (44.1)	NE	1.2 (1.1–1.4)	Questionnaire	Early (baby) swimming may be related to wheezing up to the age of 18 months.
Nystad et al.^1^	NE (38.1)	NE (38.4)	NE	1.0 (0.9–1.1)	Questionnaire	Early (baby) swimming may be related to wheezing up to the age of 18 months.
Schoefer et al.^2^	63 (9.7)	07 (3.7)	NE	NE	Doctor	It is not concluded whether baby swimming is safe in terms of atopic diseases.
Irahara et al.^16^	16 (12.7)	163 (16.8)	0.7 (0.4–1.2)	0.8 (0.4–1.6)	Questionnaire (ISAAC)	There is no evidence that swimming school attendance has a positive impact on the development of childhood wheezing or rhinitis.
Voisin et al.^14^	16 (8.2)	15 (6.4)	NE	NE	Doctor and questionnaire (ISAAC)	Swimming during childhood is associated with an increased risk of bronchiolitis, resulting in increased risk of asthma and allergic sensitization.
Bernard et al.^18^	7 (16,3)	23 (7.7)	2.3 (0.9–5.8)	2.2 (0.8–6.5)	Doctor and 15% reduction in post-exercise FEV1	Infant swimming appears to predispose children to the development of asthma and recurrent bronchitis.
Font-Ribera et al.^14^	37 (6.1)	NE	NE	1.1 (0.7–1.7)	Questionnaire (ISAAC)	Swimming in an indoor (chlorinated) pool does not increase the risk of asthma or wheezing in school-age children.

OR: odds ratio; RR: relative risk; CI: confidence interval; NE: not specified; ISAAC: International Study of Asthma and Allergies in Childhood.

 The methodological quality of the studies is summarized in Table 4, with a high risk of bias observed in questions 3, 6, and 8, and a low risk in questions 1, 4, and 7 of the Cochrane’s Bias Methods Group tool.^20^

**Table 4:** Results and conclusions on the association between early swimming and the development of asthma-related respiratory symptoms.

Item	Low risk of bias	High risk of bias	Uncertain
1. Was the selection of exposed and unexposed cohorts drawn from the same population?	6		
2. Can we trust exposure assessment?	3	2	1
3. Can we be sure that the outcome of interest was not present at the start of the study?		6	
4. Did the study match exposed and unexposed variables for all variables that are associated with the outcome of interest, or did the statistical analysis adjust for these prognostic variables?	6		
5. Can we trust the assessment of the presence or absence of prognostic factors?	2	4	
6. Can we trust the evaluation of results?		6	
7. Was the follow-up of the cohorts adequate?	6		
8. Were the co-interventions similar between the groups?		6	

### Meta-analysis

**Figure 2:**
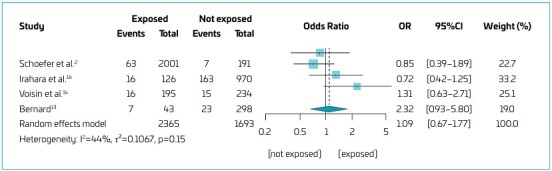
Forest plot graphic.

 The results of the meta-analysis are presented in the forest plot (Figure 2). A total of 4058 subjects, with 310 asthma events, were included in the analysis (2365 in the exposure group and 1693 in the control group). The combined effect, expressed as OR for asthma across all included studies, was 1.09 (95%CI 0.67–1.77, p=0.740). The individual studies showed a moderate level of heterogeneity (Q=5.31; df=3; p=0.150; I^2^=43.5%). 

## DISCUSSION

 This systematic review with meta-analysis found no association between childhood swimming and the risk of asthma in children and adolescents. Considering the conflicting results among the evaluated articles, which may stem for significant methodological diversity — particularly regarding study type, exposure and outcome classification, and high risk of bias — we exercise caution in our conclusions.^2,14,16,18^ We highlight the need for more robust research in this area. 

 Despite these findings, it is essential to encourage children and adolescents to engage in physical activity, including swimming, which is considered a safe and beneficial sport.^15,24-31^ Conducting research that follows children from birth to the onset of asthma presents challenges for researchers worldwide.^1,2,14-16,18^ Factors complicating this include controlling for variables such as age at exposure onset, activity frequency and duration, environmental influences, genetic predispositions, and potential recall biases from parents/guardians. 

 The World Health Organization has published guidelines addressing general health hazards related to recreational water activities.^4,32^ Nystad et al.^1^ examined the association between asthma at 18 months and early swimming among 7889 children. While a potential causal link was suggested, the findings primarily indicated that genetic factors may modulate this relationship, particularly in children with a maternal history of atopy.^6,33^ This reinforces the hypothesis that genetic predisposition plays a critical role in asthma pathogenesis, warranting further exploration of gene-environment interactions.^6,33^ Similarly, Schoefer et al.^2^ failed to reach definitive conclusions regarding children who swam before the age of 1 and were assessed at age 6. The lack of consistency in diagnostic criteria between studies, such as the medical evaluations used by Schoefer et al.^2^ compared to the parental questionnaires in Nystad et al.,^1^ may partially explain these discrepancies. 

 Voisin et al.^14^ associated childhood swimming with a higher risk of bronchiolitis, which may indirectly increase asthma and allergic sensitization risks among children who swam before age 2 and were evaluated at ages 5 and 6. This finding highlights the potential role of environmental factors, such as chlorination by-products or inadequate ventilation in indoor pools, in shaping respiratory outcomes. Addressing these environmental variables is essential to mitigating potential health risks associated with swimming. In contrast, Bernard et al.^18^ did not clarify the link between swimming from birth to age 1 and increased asthma risk between ages 10 and 13, concluding that swimming only appears to predispose children to asthma and recurrent bronchitis. 

 In contrast to studies suggesting increased risks, research by Font-Ribera et al.^15^ and Irahara et al.^16^ demonstrated that swimming in chlorinated pools does not significantly increase the risk of asthma or wheezing in school-aged children. These studies, which utilized standardized International Study of Asthma and Allergies in Childhood (ISAAC) questionnaire, underscore the potential protective or neutral effects of early swimming exposure, particularly when confounding factors such as atopic history and pool environment are well-controlled. 

 Encouraging physical activity, especially swimming, is vital for children due to its numerous benefits, including cardiovascular health, socialization, and potential as an adjunct treatment for asthma.^3,4,8,13,15,24,27-29,34,35^ A previous meta-analysis assessed the relationship between childhood swimming frequency and asthma development, concluding that attendance at swimming pools does not increase rates of physician-diagnosed childhood asthma. The review included studies with varying objectives, such as Kohlhammer et al.,^10^ which examined swimming and allergic rhinitis, and Lévesque et al.,^11^ which compared asthma development between swimmers and soccer players aged 8 to 22 years. Emerging evidence underscores the potential of swimming as a therapeutic intervention for children with asthma, offering multidimensional benefits. A recent systematic review and meta-analysis highlighted that swimming enhances quality of life and promotes physical, physiological, and emotional well-being.^12^

 In line with these findings, the last Cochrane systematic review on the topic provided further evidence on the benefits of swimming in children and adolescents with stable asthma. The review found that swimming improved pulmonary function with moderate evidence, enhanced cardiorespiratory capacity with high evidence, and was well tolerated without significant adverse effects on asthma control.^36^

 Despite similar findings,^3^ the present study focused specifically on swimming practice rather than frequency regarding asthma risk and employed different selection criteria for included articles. A limitation of this study is the small number of included articles, and the heterogeneity of age groups in the included studies may introduce confounding bias, which may lead to low statistical power and significant methodological differences. However, rigorous inclusion criteria were applied to homogenize the publications as much as possible. Nonetheless, this review’s strengths include an extensive search across major electronic databases using Mesh terms, data collection conducted by two independent researchers, and verification of findings to ensure result reliability. While the calculated odds ratios presented are acceptable, potential bias and overestimation in the meta-analysis results exist. Factors compromising methodological quality included a high risk of bias in questions 3, 6, and 8 of the Cochrane’s Bias Methods Group tool, as no study met these criteria.^20^ Still, we included articles with the most comparable characteristics and calculated heterogeneity, remaining cautious in our conclusions. 

 Children and adolescents should consistently be encouraged to engage in physical activity.^15,24-29^ The Brazilian Society of Pediatrics (*Sociedade Brasileira de Pediatria* – SBP) recommends swimming for children from six months of age, once the auditory canal is mature and appropriate safety measures are in place. The SBP advises an exposure limit of 30 minutes for this age group due to thermal regulation concerns and potential chlorine exposure, emphasizing the importance of supervision by a qualified professional. 

 Wheezing infants, particularly those with a family history of atopy, who wish to participate in swimming should seek specialized guidance and inform instructors to minimize health risks and optimize treatment when necessary.^1,2^ Pools treated with ozone, ultraviolet light, and copper and silver ionization can serve as excellent alternatives when available. 

 In conclusion, this study found no association between childhood swimming in chlorinated pools and the risk of developing asthma in children and adolescents. Any identified association was inconsistent upon meta-analysis review. These results highlight the lack of scientific evidence and the need for well-designed studies on this topic. 
